# Combining Hyperbranched and Linear Structures in Solid Polymer Electrolytes to Enhance Mechanical Properties and Room-Temperature Ion Transport

**DOI:** 10.3389/fchem.2021.563864

**Published:** 2021-06-25

**Authors:** Benxin Jing, Xiaofeng Wang, Yi Shi, Yingxi Zhu, Haifeng Gao, Susan K. Fullerton-Shirey

**Affiliations:** ^1^ Department of Chemical and Biomolecular Engineering, University of Notre Dame, Notre Dame, IN, United States; ^2^ Department of Chemical Engineering and Materials Science, Wayne State University, Detroit, MI, United States; ^3^ Department of Chemistry and Biochemistry, University of Notre Dame, Notre Dame, IN, United States; ^4^ Department of Electrical Engineering, University of Notre Dame, Notre Dame, IN, United States; ^5^ Department of Chemical and Petroleum Engineering, University of Pittsburgh, Pittsburgh, PA, United States

**Keywords:** solid polymer electrolyte, lithium ion battery, polyethylene oxide, hierarchically hyperbranched polymers, low crystallinity

## Abstract

Polyethylene oxide (PEO)-based polymers are commonly studied for use as a solid polymer electrolyte for rechargeable Li-ion batteries; however, simultaneously achieving sufficient mechanical integrity and ionic conductivity has been a challenge. To address this problem, a customized polymer architecture is demonstrated wherein PEO bottle-brush arms are hyperbranched into a star architecture and then functionalized with end-grafted, linear PEO chains. The hierarchical architecture is designed to minimize crystallinity and therefore enhance ion transport *via* hyperbranching, while simultaneously addressing the need for mechanical integrity *via* the grafting of long, PEO chains (*M*_*n*_ = 10,000). The polymers are doped with lithium bis(trifluoromethane) sulfonimide (LiTFSI), creating hierarchically hyperbranched (HB) solid polymer electrolytes. Compared to electrolytes prepared with linear PEO of equivalent molecular weight, the HB PEO electrolytes increase the room temperature ionic conductivity from ∼2.5 × 10^–6^ to 2.5 × 10^−5^ S/cm. The conductivity increases by an additional 50% by increasing the block length of the linear PEO in the bottle brush arms from *M*_*n*_ = 1,000 to 2,000. The mechanical properties are improved by end-grafting linear PEO (*M*_*n*_ = 10,000) onto the terminal groups of the HB PEO bottle-brush. Specifically, the Young’s modulus increases by two orders of magnitude to a level comparable to commercial PEO films, while only reducing the conductivity by 50% below the HB electrolyte without grafted PEO. This study addresses the trade-off between ion conductivity and mechanical properties, and shows that while significant improvements can be made to the mechanical properties with hierarchical grafting of long, linear chains, only modest gains are made in the room temperature conductivity.

## Introduction

Solid polymer electrolytes based on polyethylene oxide (PEO) have been widely explored to replace liquid or gel electrolytes in rechargeable Li-ion batteries. In addition to inhibiting dendrite growth, the solid polymer has the potential to replace relatively volatile plasticizers, offering the promise for a greener battery. Although pure PEO has a flexible, linear backbone and a glass transition temperature (T_g_) of −60°C, ion transport within PEO is slow at room temperature. For example, the ionic conductivity of (PEO)_10_:LiClO_4_ is on the order of 10^–6^ S/cm Croce et al. (1998); Reddy et al. (2006), which is three orders of magnitude lower than required (Shin et al., 2003). One problem with PEO-based electrolytes is their tendency to recrystallize, which inhibits segmental mobility and decreases ionic conductivity. The recrystallization time can vary widely depending on ion concentration and water content. For example, it can take up to 3 days for the electrolyte (PEO)_8_:LiClO_4_, to recrystallize when the water concentration in the electrolyte is <1 wt% (Fullerton-Shirey et al., 2011). To prevent recrystallization of the polymer and increase the segmental mobility, plasticizers are commonly added (Golodnitsky et al., 1996; Michael et al., 1997; Song et al., 1999). However, the amount of plasticizer required to increase the conductivity to 10^–3^ S/cm also compromises the mechanical properties such that the polymer electrolyte is no longer a free-standing, solid film.

Two popular approaches to increase conductivity without compromising the mechanical properties are to add metal oxide nanoparticles Krawiec et al. (1995); Lin et al. (2018); Zhang et al. (2018), or ionic liquids to the polymer host (Shin et al., 2003; Zhan et al., 2019). Metal oxide nanoparticles improve the mechanical properties; however, they only increase the conductivity by one order of magnitude (Krawiec et al., 1995; Croce et al., 1998). Ionic liquids have a plasticizing effect—they decrease crystallinity and increase the ionic conductivity (Chaurasia et al., 2011; Kumar et al., 2011). Another approach is to directly tailor the polymer architecture, for example by hyperbranching the PEO Gao and Yan (2004); Lapienis (2009) to inhibit crystallization *via* the addition of branching units that disrupt chain packing (Hawker et al., 1996). Several different molecular architectures of PEO polymers, including comb-brush or bottle-brush (Jannasch, 2000; Zhang et al., 2004; Higa et al., 2005; Uno et al., 2008; Yoshimoto et al., 2009; Barteau et al., 2013; Li et al., 2014; Daigle et al., 2015; Jing and Evans, 2019; Li et al., 2019; Rosenbach et al., 2019), chemically or physically crosslinked bottle-brush Shim et al. (2014) star Li et al. (2008); Niitani et al. (2009); Kim et al. (2012), and other highly branched (i.e. hyperbranched) structures Lee et al. (2011) have been developed as solid polymer electrolytes with enhanced room-temperature conductivity. Briefly, PEO chains below the entanglement length (*M*_n_ ∼ 1,700) Fetters et al. (2007) were used in these studies to increase the amorphous fraction; however, lowering crystallinity with short polymer chains without entanglement often compromises the mechanical properties (Ho et al., 2003; Quartarone and Mustarelli, 2011).

**SCHEME 1:**
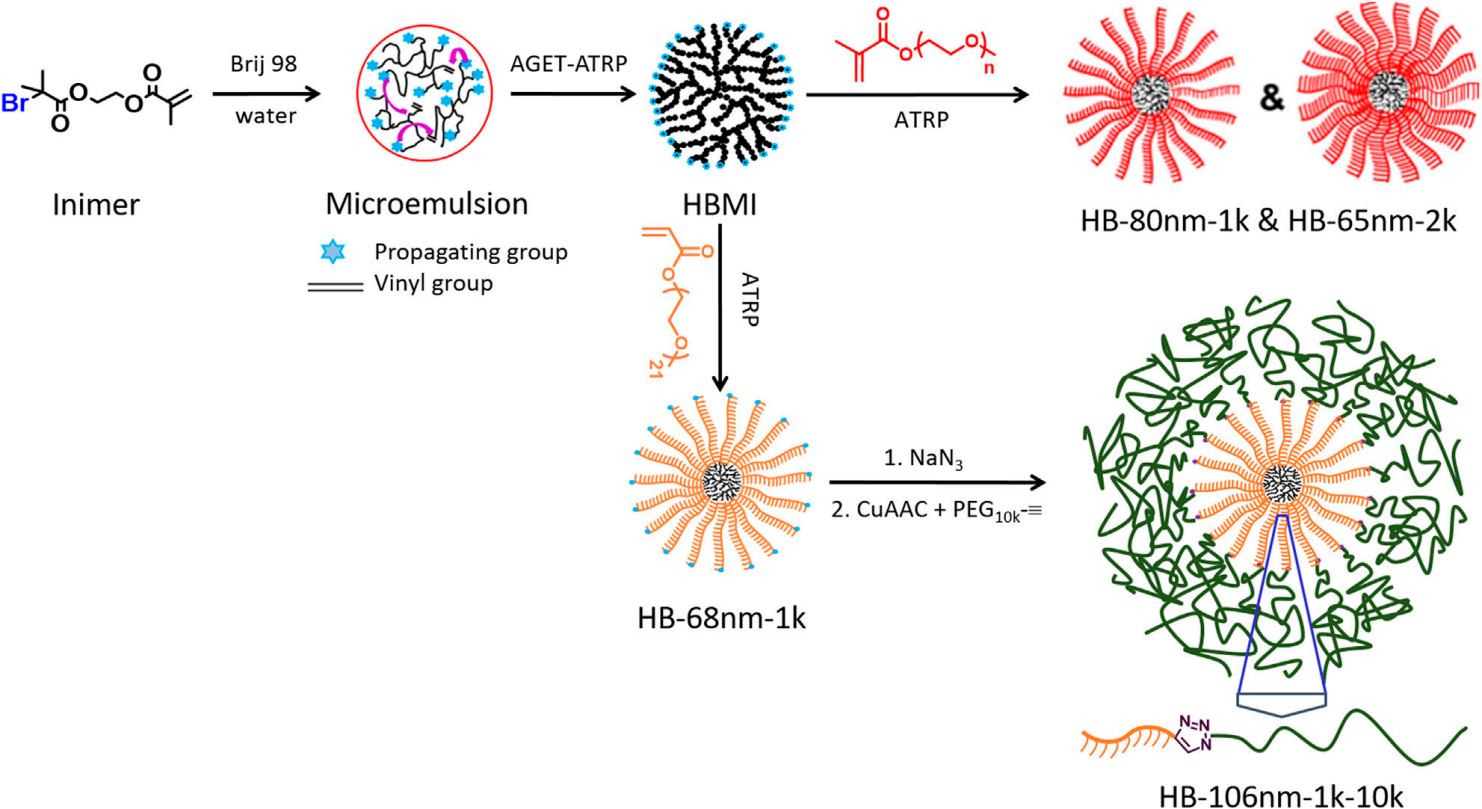
Schematic of the synthesis routes of HB PEO (HB-1k; HB-2k) and end-capped HB PEO (HB-1k-10k).

In this work, we integrate hyperbranched (HB) polymers with long, linear, grafted polymers (*M*_*n*_ = 10,000) to create a hierarchical HB structure. We address the need for enhanced ion transport by inhibiting crystallization *via* hyperbranching while simultaneously retaining mechanical integrity *via* high molecular weight chains. Specifically, we designed and synthesized two types of hierarchical HB, PEO-based polymers, illustrated in Scheme 1. One type is a hyperstar PEO polymer containing a HB core and bottle-brush arms for which the PEO side chains have *M*_n_ = 1,000 and 2,000, the latter of which is above the entanglement molecular weight of PEO. The second type is similar to the first; however, long, linear PEO chains are grafted onto the terminal groups of bottle-brush arms, which is expected to further improve the entanglement between HB PEO polymers and improve the mechanical properties. Both types of polymers involve the graft-from method to grow bottle-brush arms from a multifunctional macroinitiator (MI).

Electrolytes consisting of hierarchically HB PEO combined with lithium bis(trifluoromethane)sulfonimide (LiTFSI) in an ether oxygen (EO) to lithium ion molar ratio of 25:1 are measured. LiTFSI was chosen as the salt for its high dissociation ratio Edman (2000), low anion mobility, and high Li-ion transport number (Quartarone and Mustarelli, 2011; Stolwijk et al., 2012). The effect of polymer architecture on the crystallization and ionic conductivity was measured by atomic force microscopy (AFM), differential scanning calorimetry (DSC), and impedance spectroscopy. The HB PEO electrolytes are benchmarked against linear PEO electrolytes with the same salt concentration, and the results show that the hierarchically HB polymer architecture has a stronger impact on the mechanical properties than the ionic conductivity.

## Materials and Methods

### Materials

The following chemicals and solvents were purchased from Aldrich with the highest available purity and used as received unless otherwise stated: 4,4′-dinonyl-2,2′-dipyridyl (dNbpy), *N,N,N′,N″,N″*-pentamethyldiethylenetriamine (PMDETA), CuBr_2_, sodium ascorbate, 2-bromoisobutyryl bromide, 2-hydroxyethyl methacrylate, poly (ethylene glycol) methyl ether (*M*_n_ = 10,000, L10k), polyoxyethylene (20) oleyl ether (Brij98), dichloromethane (DCM) and LiTFSI (puriss, 99.95%). Inimer 2-([2-bromoisobutyryloxyethyl methacrylate] [BIEM]) was synthesized by a one-step reaction of 2-bromoisobutyryl bromide with 2-hydroxyethyl methacrylate (Matyjaszewski et al., 1997; Min and Gao, 2012; Graff et al., 2015). PEO macromonomers, including oligo (ethylene glycol) methyl ether methacrylate (OEGMA, *M*_n_ = 950 [L1k] and 2,000 [L2k]) and oligo (ethylene glycol) methyl ether acrylate (OEGA, *M*_n_ = 1,000 [L1kA]), were purchased from Aldrich and passed through aluminum column to remove inhibitors before use. Linear PEO of *M*_n_ = 1.5 × 10^6^ and polydispersity M_w_/M_n_ = 1.13 (L1.5M), was purchased from Agilent Technologies and used directly.

### Synthesis of Hyperbranched Polyethylene Oxide

HB PEO was synthesized following the procedures illustrated in Scheme 1 (also detailed in the Supporting Information). Briefly, HB MIs, which contain about 1,180 bromine initiating sites, were used for the polymerization of PEO-based OEGMA (L1k and L2k) and OEGA (L1kA) to generate HB bottle brushes. To generate a hierarchically HB structure, the HB bottle brushes were further modified by end-capping each bottle-brush arm with a linear PEO, L10k. The products of different hierarchically HB PEO polymers were named as “HB-x-y,” in which “x” represents the measured hydrodynamic diameter (*D*_h_) of the HB PEO and “y” represents the molecular weight (*M*_n_) of the PEO side chains in bottle brushes. For instance, HB-65nm-2k is a HB PEO polymer with *D*_h_ = 65 nm in DCM, that includes OEGMA L2k as PEO bottle brushes. Two HB PEOs with different side-chain *M*_n_ were synthesized, namely HB-65nm-2k and HB-80nm-1k, in two steps as illustrated in Scheme 1: First, HB MI was synthesized using activators generated by electron transfer (AGET)-atom transfer radical polymerization (ATRP) of AB*-type inimer, which contains initiator fragment B* and monomer vinyl group A in one molecule, BIEM, in microemulsion Matyjaszewski et al. (1997); Min and Gao (2012); Graff et al. (2015) (see details in Supporting Information). The produced HB MI was then purified and used as MI to initiate the ATRP of macromonomers to introduce PEO bottle-brush arms.

To improve the mechanical properties of HB PEO, linear L10k was grafted to end-cap HB-68nm-1k to enhance the entanglement between PEO chains from different HB PEO polymers. The produced hierarchically HB PEO has a hydrodynamic diameter D_h_ = 106 nm and is denoted as HB-106nm-1k-10k. For its synthesis, the bromine group at each bottle-brush arm end was replaced by azido group and followed by clicking alkyne-functionalized L10k to the end.

### Characterization

The molecular weights of polymers were measured by size exclusion chromatography (SEC) with dimethylformamide (DMF) as the mobile phase. The DMF SEC used Polymer Standards Services (PSS) columns (guard, 10^4^, 10^3^, and 10^2^ Å Gram 10 columns) at 55°C with flow rate = 1.00 ml/min and connected with a RI detector (Waters, 2,410) using PSS WinGPC 7.5 software. The apparent number-average molecular weight (M_n,RI_) of HB MI was calculated based on linear poly (methyl methacrylate) (PMMA) standards and the M_n,RI_ values of HB PEO polymers were calculated based on linear PEO standards. The absolute M_n_ of HB MI core was measured from SEC in tetrahydrofuran (THF) mobile phase equipped with PSS columns (guard, 10^5^, 10^3^, and 10^2^ Å SDV columns) and a multi-angle laser light scattering (MALLS) detector (Wyatt Technology, DAWN HELEOS II) at the light wavelength of 658 nm and analyzed by ASTRA software (Wyatt Technology) with the differential index of refraction, dn/dc of 0.084 ml/g for polymethacrylate-based polymers (Gao et al., 2005; Sumerlin et al., 2005). The measurements were conducted at constant temperature at 35°C and THF flow rate of 1.00 ml/min.

Nuclear magnetic resonance (NMR) spectroscopy was carried out on a Bruker 500 MHz spectrometer operated in the Fourier transform mode at 25°C (Supplementary Figures S1A–C). FT-IR spectra of HB-68nm-1k and HB-106nm-1k-10k were acquired using a FT/IR-6300 (JASCO) (Supplementary Figure S1D). The D_h_ and coefficient of variation (CV) of all the samples in DCM were determined using dynamic light scattering (DLS) equipped with a Zetasizer Nano-ZS (Malvern Instruments, Malvern, United Kingdom) at the He-Ne laser wavelength of 633 nm.

### Solid Polymer Electrolyte Preparation

Due to the limited amount of HB PEO from synthesis, freeze-drying followed by hot-pressing for conductivity measurements and spin-coating a thin film for AFM are used. Solid electrolytes were obtained by mixing HB PEO with LiTFSI in deionized water (Barnstead Nanopure II) at an EO:Li molar ratio of 25:1. This solution was freeze-dried (Labconco Freezone 4.5 Freeze Dryer) and stored at 120°C in vacuum for 72 h to remove water. The linear chain PEO electrolytes were prepared at the same salt concentration using the same method.

### Differential Scanning Calorimetry

The thermal properties of the electrolytes were measured using a TA Instrument Q2000 DSC under continuous nitrogen purge. The accuracy of temperature control is ± 0.01°C and the measurement sensitivity is 0.2 µW. Samples of 8–10 mg were placed in aluminum pans and dried in a vacuum oven at 110°C for 72 h. The pans were immediately hermetically sealed after being removed from the vacuum oven. Samples were first heated from room temperature to 120°C at 20°C/min and held for 10 min to remove the thermal history. Then the samples were cooled from 120 to −90°C at 5°C/min to obtain crystallization temperature, T_c_ and crystallization enthalpy, ΔH_c_. After equilibrium at −90°C for 10 min, the samples were heated to 100°C at 10°C/min to determine the glass transition temperature, T_g,_ melting temperature, T_m_, and melting enthalpy, ΔH_m_. According to the phase diagram for PEO:LiTFSI Edman et al. (2000), pure PEO is the crystalline phase that will form at EO:Li = 25:1. Therefore, the crystal fraction, X_crystal_ of the PEO/LiTFSI electrolytes can be calculated by dividing the measured ΔH_c_ or ΔH_m_ (normalized by the mass of the electrolyte) by 203 J/g, which is the melting enthalpy of a perfect PEO crystal (Wunderlich, 1980). We found nearly no difference in the X_crystal_ calculated from either ΔH_c_ or ΔH_m_ for all the electrolytes in this work, suggesting the heating rate of 10°C/min and the cooling rate of 5°C/min are sufficiently slow to allow PEO samples to achieve the highest crystallinity.

### Atomic Force Microscopy

The morphological structure of neat HB PEO samples was characterized by AFM (Bruker Nano Multimode 8, Nanoscope IV Controller) operated in tapping mode with a silicon probe (Bruker Nano, TEPSAW). The samples were deposited on a polished silicon wafer (Silicon Quest International), which was cleaned by piranha solution at 120°C for 1 h. To deposit the polymer, 0.01 g/L HB PEO in acetonitrile was spin-coated (Laurell Technoloiges, WS-400BZ-6NPP/LITE) onto the wafer at 8,000 rpm, yielding a ∼10 *µ*m thick film as measured by an AFM step height scan.

To determine the Young’s modulus, nano-indentation measurements were conducted using PeakForce Mode with the same AFM. Because Young’s modulus measurements require the spring constant of the AFM probe to match that of the sample, several AFM probes with spring constant ranging from 0.5–45 N/m were used to select appropriate probes. The spring constant of selected AFM probes (Bruker Nano TEPSAW or ScanAsyst-Air) were calibrated by the thermal tune method Langlois et al. (2007), and the force curves were fitted by the Sneddon model to yield Young’s modulus using the Nanoscope Analysis software (Sneddon, 1965). For nano-indentation, HB PEO films with thickness ∼10 µm were prepared on a clean Si wafer by drop-casting of 10 g/L HB PEO solution and air-dried. The samples were subsequently placed in a vacuum oven at 50°C for 24 h to remove the solvent. Because the typical indentation depth in this work is below 100 nm after carefully selecting an AFM probe with matched spring constant, the ∼10 µm thick film is sufficiently thick to eliminate any effect of the substrate on Young’s modulus because it is below the Bueckle’s indentation depth limit (i.e. 10% of specimen thickness) (Persch et al., 1994).

### Conductivity Measurements

To prepare the parallel plate samples for impedance measurements, the electrolyte was placed on a stainless-steel electrode, confined inside of a Teflon O-ring, and dried in vacuum oven at 110°C for 72 h to drive off residual solvent. The top electrode was positioned, and the parallel plate structure was hot-pressed to produce a uniform film of diameter 15 mm and thickness between 100 and 200 μm, which was determined by a micrometer screw gauge.

Electrical measurements were made using a Cascade Summit 11861 probe station equipped with a temperature controller (ThermoChuck, Temptronic TP03000A-X300) to control the temperature from 22 to 100°C with ± 0.1°C accuracy. In this work, all the electrolytes were measured after 10 min of thermal equilibrium at each temperature in a nitrogen environment. Impedance was measured using an Agilent, 4294A impedance analyzer over a frequency range of *ω* = 40 Hz–110 MHz with an AC-voltage of 500 mV and no DC bias. The conductivity,  σ(ω) was calculated from the measured Z(*ω*) using Eq. 1:

$$\sigma \left(\omega \right)=\frac{d\cos\theta \left(\omega \right)}{A\left|Z\left(\omega \right)\right|}$$ (1)

where ***d*** is the electrolyte thickness, ***A*** is the electrode surface area, |Z(*ω*)| is the magnitude of the measured impedance, and ***θ*** is the measured phase angle. The conductivity was obtained from the plateau value of σ(ω)  (*i.e.* the region over which the conductivity is frequency independent). In addition to the HB electrolytes, a control measurement was made on linear PEO (M_w_ = 600,000 g/mol)/LiClO_4_ at EO:Li = 10:1 with the same sample preparation procedure described above. The conductivity of linear PEO/LiClO_4_ was 3.0 × 10^–7^ S/cm at 22°C, which agrees well with the previously reported value Fullerton-Shirey and Maranas (2009); Schaetzl et al. (2014), suggesting that the sample preparation conditions result in samples with similar properties to previous reports.

## Results and Discussion

**FIGURE 1:**
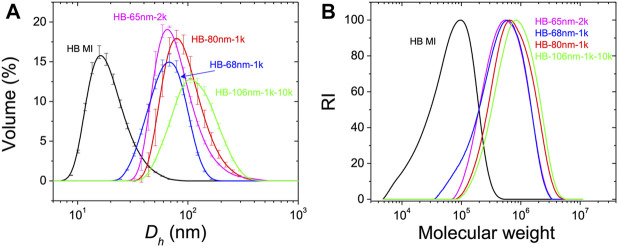
**(A)** Volume fraction distribution of the measured hydrodynamic diameter, *D*_h_, of HB MI and HB PEO in DCM by DLS. **(B)** SEC trace profiles of HB MI and HB PEO in DMF based on linear PMMA standards for HB MI and linear PEO standards for HB PEO samples.

The hydrodynamic diameter, D_h_ of HB MIs and HB PEO polymers were measured in DCM by DLS. As shown in Figure 1, and tabulated in Table 1, the measured D_h_ of HB MI is 13 nm. After chain extension by the ATRP of macromonomers from the HB MI, the D_h_ increases to 65, 68, and 80 nm for HB PEOs incorporating PEO bottle-brushes of varying arm lengths (HB-65nm-2k, HB-68nm-1k, and HB-80nm-1k, respectively). The synthesis of HB-106nm-1k-10k was accomplished after an additional grafting-onto reaction to cap the bottle-brush arm ends of HB-68nm-1k with alkyne-terminated linear PEO chain (M_n_ = 10,000). The D_h_ of HB-106nm-1k-10k was 106 nm (increased from 68 nm), confirming the successful grafting of the linear PEO onto the HB-68nm-1k. The measured molecular weights and the polydispersity of polymers by SEC are shown in Figure 1B, and tabulated in Table 1. For the HB MI core, the apparent number-average molecular weight based on linear PMMA standards in SEC with DMF as mobile phase is M_n_ = 132,000, which is lower than its absolute value M_n_ = 328,000, determined by MALLS detector coupled in THF SEC system, suggesting a high degree of branching (DB) in the HB MI. Indeed, the DB was confirmed by ^1^H NMR spectroscopy of the purified HB MI as DB = 0.27 (i.e. one dendritic unit per every 7.5 structural units in the HB MI structure). Based on the absolute molecular weight, one HB MI contains 1,180 Br initiating sites on average that can initiate the ATRP of OEGMA monomers to produce HB PEO. The apparent M_n_ of the HB PEO increases with chain extension of varied bottle-brush arm length; however, the absolute M_n_ of HB PEO cannot be obtained because the value exceeds the detection range of column-based chromatography technique (Makan et al., 2012). Based on these data, we assume that the absolute molecular weight is approximately three times larger than the apparent molecular weight. The apparent molecular weights and sizes for all the HB PEO samples used in this work are summarized in Table 1.

**TABLE 1:** Structural information for the HB MI and HB PEO synthesized in this work.

Polymer	*D*_h_ (nm)^a^	CV^a^	*M*_n_,_RI_ ^b^	*M*_w_/*M*_n_
HB MI (core)	13	0.25	132 × 10^3^	1.3
HB-65nm-2k	65	0.23	452 × 10^3^	1.2
HB-68nm-1k	68	0.21	478 × 10^3^	1.29
HB-80nm-1k	80	0.19	621 × 10^3^	1.29
HB-106nm-1k-10k	106	0.17	913 × 10^3^	1.30

a Hydrodynamic diameter (*D*_h_) and coefficient of variation (CV) in DCM measured by DLS.

b Apparent number-average molecular weight, *M*_n,RI_ and polydispersity *M*_w_/*M*_n_ measured by DMF SEC, calibrated with linear PMMA standards.

**FIGURE 2:**
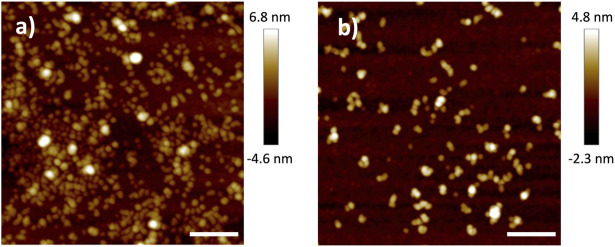
AFM micrographs showing the topology of **(A)** HB-68nm-1k and **(B)** HB-106nm-1k-10k, both of which were deposited from their corresponding dilute (0.01 g/L) HB PEO solution in acetonitrile. The scale bar for both panels is 200 nm.

Before characterizing the polymer electrolyte film, the shape and size of the HB PEO was characterized on Si substrates by tapping mode AFM. Dilute solutions of the material (0.01 g/L HB PEO in acetonitrile) were deposited on a Si substrate *via* spin-coating. The resulting low coverage sample has a particle-like morphology, shown in Figure 2 by AFM scans in tapping mode. The HB-68nm-1k have an average diameter of 30 nm and average height of 2.6 nm. After grafting with L10k, the diameter of HB-106nm-1k-10k remains similar to that of HB-68nm-1k, but the height increases to approximately 3.5 nm, on average, suggesting a ∼35% volume expansion. While the volume change measured by AFM is smaller than that measured by DLS and SEC, this is likely due to inhomogeneous swelling of the surface-confined polymers. Regardless, AFM confirms the increasing polymer size with additional grafting.

**FIGURE 3:**
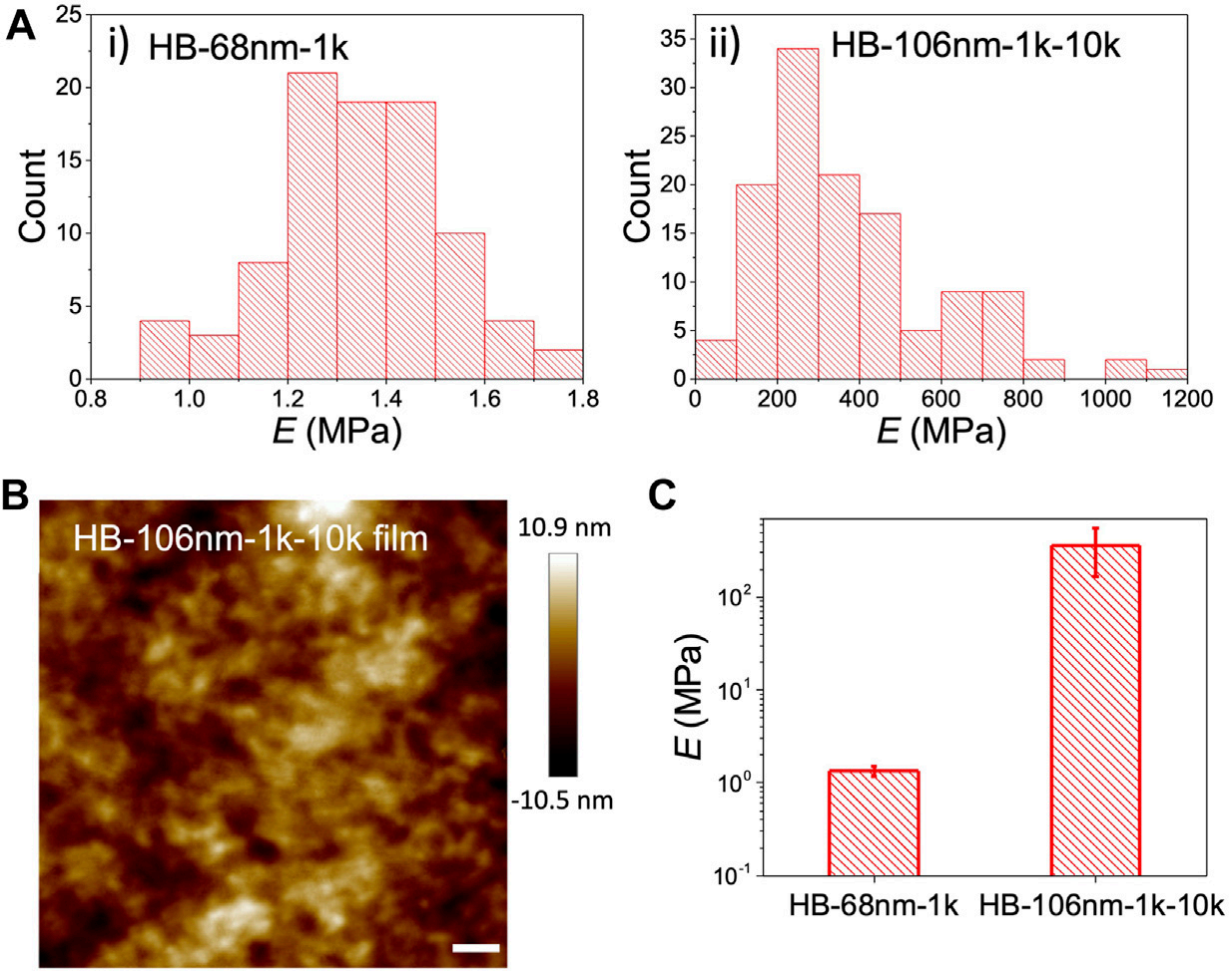
**(A)** Distribution of Young’s modulus measurements by nano-indentation with AFM for i) HB-68nm-1k and ii) HB-106nm-1k-10k. (B) AFM topology scans of the HB-106nm-1k-10k film deposited from concentrated (10 g/L) HB PEO solution in acetonitrile. The scale bar is 200 nm. **(C)** Measured Young’s modulus (*E*) of HB-68nm-1k and HB-106nm-1k-10k films by nano-indentation with AFM, which was averaged over 100 force curve measurements shown in **(B)**. The error bars represent one standard deviation from the mean.

Moving onto the solid electrolyte films, the Young’s modulus (*E*) of the HB PEO films was determined by AFM nano-indentation over 100 force-distance measurements to ensure statistical significance, as shown in Figure 3A. (Representative force-distance curves are provided in the Supplementary Figure S2). Samples were prepared by drop-casting a concentrated HB PEO solution (10 g/L) onto Si substrate to give a continuous film. It is noted that the topology of HB-68nm-1k film cannot be characterized by AFM because the bulk HB-68nm-1k polymer is a viscous fluid Lee et al. (2011) and is too soft even for the softest AFM probe. In contrast, the morphology of the HB-68nm-1k-10k film capped with PEO chains, can be readily measured by AFM (Figure 3B), demonstrating the significantly enhanced mechanical properties. No crystal domains are detected in the HB-106nm-1k-10k—at least by AFM—in contrast to the crystalline morphology of the linear L10k PEO samples (Supplementary Figure S3). This observation is also confirmed by DSC, presented below. More importantly, the measured Young’s moduli of HB PEO polymers differ considerably, as shown in Figure 3C: the *E* of HB-106nm-1k-10k is ∼358 MPa, which two orders of magnitude higher than that of HB-68nm-1k, and is comparable to that of linear PEO with high M_n_ and crystallnity (Jee et al., 2013). Thus, the moduli measurements confirm that grafting L10k onto the outside of the HB PEO significantly improves the mechanical properties.

**FIGURE 4:**
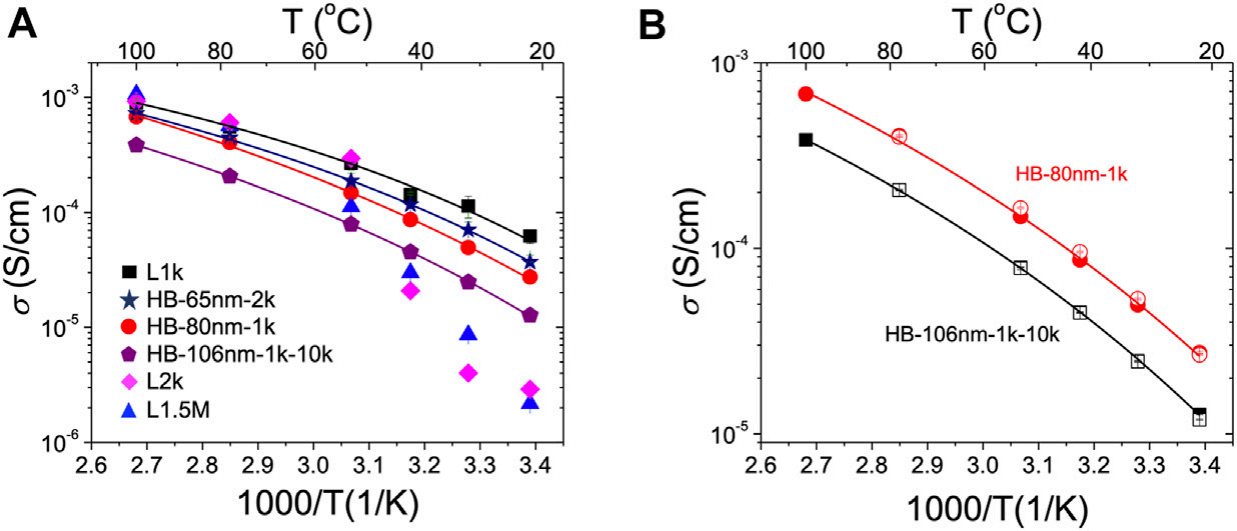
Temperature-dependent ionic conductivity showing the effect of HB PEO molecular architecture and thermal history. Measured conductivity (*σ*) vs. 1,000/T (K^−1^) of **(A)** HB and linear PEO electrolytes and **(B)** HB-80nm-1k (circles) and HB-106nm-1k-10k (squares) upon heating (filled symbols) and cooling (open symbols). The solid lines in **(A)** and **(B)** are fits using the Vogel-Tamman-Fulcher Eq. 2. Samples are equilibrated at each temperature for 10 min before data collection.

The temperature-dependent ionic conductivity (*σ*) of the HB-80nm-1k, HB-65nm-2k, and HB-106nm-1k-10k electrolytes (EO:LiTFSI = 25:1) were measured and compared to that of their corresponding PEO components, L1k, L2k, and L1.5M, with the equivalent salt concentrations. Room temperature impedance, phase angle and conductivity data are provided vs. frequency in Supplementary Figure S4; the calculated conductivity values are plotted vs. inverse temperature in Figure 4A for all samples. Distinct from the previous studies with electrolytes based on only linear PEO, the conductivity of the HB PEO electrolytes shows a weaker dependence on temperature. Specifically, the conductivity decreases gradually with decreasing temperature as opposed to the linear PEO electrolytes (L2k and L1.5M) that show a sharp decrease in conductivity at T < 50°C. The abrupt conductivity decrease in linear chain PEO is due to chain crystallization (Appetecchi et al., 2001; Fullerton-Shirey and Maranas, 2009). In contrast, above the melting point, both linear chain and HB PEO will be highly mobile thereby decreasing the ionic conductivity difference between the two.

The highest room temperature conductivity of the HB electrolytes is the HB-65nm-2k, with a room-temperature conductivity of ∼3.5 × 10^–5^ S/cm, which is ∼50% higher than that of HB-80nm-1k and 10 times higher than the corresponding PEO macromonomer electrolyte, L2k. For the HB-80nm-1k electrolyte, the equivalent linear chain electrolyte, L1k, does not crystallize and therefore has a higher room-temperature conductivity than the HB polymer.

In addition to comparing the low molecular weight component of the HB electrolytes to their corresponding linear macromolecule, another way to compare is between the *total* molecular weight. For example, we can compare L1.5M (total *M*_n_ = 1.5 × 10^6^) to HB-65nm-2k (apparent M_n,RI_ = 4.5 × 10^5^) and account for the three times difference between the total and apparent M_n_ as described above. In this case, the conductivity of the HB-65nm-2k electrolyte is ∼20 times higher than that of linear PEO, L1.5M. Hence, the enhanced conductivity of the HB-65nm-2k PEO electrolyte over the equivalent linear macromolecules suggests that the introduction of PEO side chains is beneficial for conductivity. However, when linear L10k PEO is grafted to the ends, the conductivity is *lower* than that of its precursor, but only by 50%.

A recent report by Li and co-workers also shows that grafted PEO side chains enhance conductivity (Li et al., 2019). Specifically, PEO side chains with number-average molecular weight (M_n_) from 300 to 950 increased room temperature conductivity up to two orders of magnitude. However, these chains remain below the entanglement molecular weight of PEO (∼1,700) Fetters et al. (2007), yielding only modest enhancement of the mechanical properties. In stress-strain measurements, the tensile stress of the M_n_ = 950 sample was 2.6 MPa—only a few percent higher than the linear PEO electrolytes with M_v_ = 100,000.

It is noted that the conductivity of the HB PEO electrolytes becomes almost indistinguishable from the linear chain counterpart at T > 60°C. One possible explanation as to why the HB conductivity is not as strongly dependent on temperature as the linear chain equivalent is that ion hopping Mao et al. (2000); Borodin and Smith (2007); Maitra and Heuer (2007); Diddens et al. (2010) is suppressed by the rigid backbone present in the HB polymer. These data show that the hierarchically HB architecture provides enhanced conductivity compared to the linear chain equivalent above the entanglement molecular weight only at temperatures less than 60°C.

Considering that crystalline regions usually exhibit lower conductivity than amorphous regions Sequeira and Santos (2010), we attribute the enhanced conductivity of the HB PEO electrolytes to their low crystallinity and slow recrystallization kinetics. For amorphous polymers, the temperature-dependent conductivity can be fit by the Vogel–Tamman–Fulcher (VTF) equation (Armand, 1983; Quartarone and Mustarelli, 2011):

$$\sigma =AT^{-0.5}e^{-B/\left(T-T_{0}\right)}$$ (2)

where *A* is a pre-exponential factor, *T*_*0*_ is the ideal glass transition temperature, and *B* is the pseudo-activation energy for the conductivity. All the conductivity data for the HB PEO electrolytes can be well fitted by the VTF equation, yielding T_0_ = 181–205 K with good accuracy (> 0.995). T_0_ is typically 10–50 K lower than the T_g_ of PEO, as predicted (Armand, 1983; Quartarone and Mustarelli, 2011). The quality of the fits suggest that the HB PEO electrolytes are primarily amorphous during the measurement. Additionally, as shown in Figure 4B, the temperature-dependent conductivities of the HB PEO electrolytes completely overlap when increasing and decreasing the temperature, indicating no thermal hysteresis. These results sharply contrast linear PEO electrolytes for which the conductivity depends strongly on the thermal history (Appetecchi et al., 2001; Fullerton-Shirey et al., 2011). Thus, the absence of thermal hysteresis in the HB PEO electrolytes also suggests low crystallinity or slow recrystallization kinetics.

**FIGURE 5:**
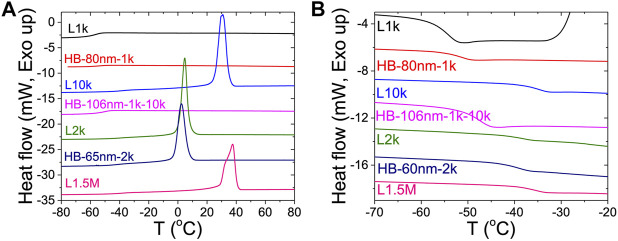
DSC measurements of heat flow for different HB and linear PEO/LiTFSI electrolytes upon **(A)** cooling from 80 to −90°C and **(B)** heating from −90 to −20°C. T_c_ is capture in panel **(A)** and T_g_ is capture in panel **(B)** and tabulated in Table 2.

DSC was used to directly measure the crystallinity of the HB PEO electrolytes. Samples were heated at 10°C/min and cooled at 5°C/min. As shown in Figure 5A, a recrystallization peak appears on cooling at T_c_ = 37.5°C for the linear PEO electrolyte, L1.5M, which is more than 35° higher than that of HB-65nm-2k (T_c_ = 2.4°C). As a reminder, both samples share equivalent total *M*_*n*_ but have different architectures. Because Tc < 22°C for HB-65nm-2k, this sample does not recrystallize during the room-temperature conductivity measurement, giving rise to the enhanced conductivity of HB-65nm-2k (Supplementary Figure S5 for additional DSC data).

The crystalline fraction, *X*_*crystal*_ *,* of linear and HB PEO electrolytes was calculated based on the DSC data and summarized in Table 2. The *X*_*crystal*_ of HB-65nm-2k is 35.5% and considerably lower than the linear chain equivalents that range from 45 to 53.5%. The highest reduction of crystallinity to 0.1% is achieved with HB-106nm-1k-10k, suggesting that crystallization of the bottle brush is inhibited by grafting linear PEO chains to the outer layer. The DSC results clearly indicate that introducing the HB chains to the architecture of the PEO electrolytes can effectively reduce the crystallinity, and the trend corresponds with increases in conductivity in Figure 4.

**TABLE 2:** Thermal properties of the electrolytes: glass transition (T_g_), melting (T_m_), crystallization temperatures (T_c_), and crystal fraction (X_crystal_) of the HB and linear PEO/LiTFSI electrolytes.

PEO electrolyte	T_g_ (^o^C)	T_m_ (^o^C)	T_c_ (^o^C)	X_crystal_ (%)
L1k	−53.9	27.5	−23.5*	26.6
L2k	−37.9	45.6	4.7	44.8
L10k	−34.6	55.0	30.4	49.4
L1.5M	−35.2	55.4	37.5	53.5
HB-80nm-1k	−51.2	28.4	1.8	2.4
HB-65nm-2k	−38.2	43.4	2.4	35.5
HB-106nm-1k-10k	−47.4	42.0	−	0.1

* Measured during heating sweep after equilibrating at −90.

o C for 10 min (Supplementary Figure S5), indicating sluggish recrystallization kinetics.

In contrast to the T_c_ values, the measured T_g_ values of the HB PEO electrolytes, except HB-106nm-1k-10k, show little change from those of their corresponding macromonomers (*e.g.* T_g_ = −53.9°C for L1k as compared to T_g_ = −51.2°C for HB-80nm-1k), indicating that the grafted PEO side chains likely experience similar PEO segmental mobility as the linear PEO chains. While the HB-106nm-1k-10k electrolyte has a T_g_ that is, intermediate between the T_g_ values of the corresponding linear polymers, only 1 T_g_ is observed −47.4°C, indicating no phase separation between the amorphous HB PEO and the linear, grafted PEO 10k arm. Considering HB-106nm-1k-10k as a miscible polymer blend, we can calculate its T_g_ as the weighted average of the individual T_g_ values corresponding to the two main components (*i.e*, $T_{g,1}$ (L10k) = −34.6°C and $T_{g,2}$ (HB-1k) = −51.2°C) using the Fox equation (Hiemenz and Timothy, 2007)

$$\frac{1}{T_{g}}=\frac{w_{1}}{T_{g,1}}+\frac{1-w_{1}}{T_{g,2}}$$ (3)

where $w_{1}$ is the weight fraction (∼26%) of L10k. The calculated T_g_ of HB-106nm-1k-10k is −46.8°C, in good agreement with the measured value.

## Conclusions

The fundamental trade-off between ionic conductivity and the mechanical properties of solid polymer electrolytes has made it difficult to improve both properties simultaneously. The approach taken in this study is to tailor the architecture of HB PEO electrolytes to create a hierarchical structure with end-grafted PEO attached to the bottle-brush core. In comparison to the linear-chain PEO electrolytes of the same molecular weight, the HB PEO electrolytes increase the room temperature conductivity by more than one order of magnitude. The conductivity can be further increased by 50% by increasing the block length of linear PEO in the HB PEO bottle-brush from *M*_*n*_ = 1,000 to 2,000. However, it is only by creating the hierarchical structure where 10k linear PEO is end-grafted onto the terminal groups of the HB PEO bottle-brushes that significant improvements are made to the mechanical properties. Specifically, Young’s modulus increases by two orders of magnitude to 358 MPa-a modulus comparable to commercial PEO. DSC results show that the grafting of the long chains to the bottle brush frustrates HB PEO crystallization, decreasing the crystal fraction to 0.1%, while only decreasing the conductivity by a factor of two. Even though the conductivity decreases slightly, it still remains one order of magnitude larger than the equivalent electrolyte with linear chain PEO. While the room temperature conductivities of these samples remain below the value needed for practical application, the hierarchical HB PEO electrolytes demonstrate that mechanical properties can be significantly improved with only a minor trade-off in the ionic conductivity.

## Data Availability

The original contributions presented in the study are included in the article/Supplementary Material, further inquiries can be directed to the corresponding authors.
