# Supplementary material for: Combining Hyperbranched and Linear Structures in Solid Polymer Electrolytes to Enhance Mechanical Properties and Room-Temperature Ion Transport
Source: Front Chem. 2021 Jun 25;9:563864. doi: 10.3389/fchem.2021.563864 (PMC8268023; doi:10.3389/fchem.2021.563864)
Supplement: Supplementary file 1 [file DataSheet1.pdf]

Supplementary Material

Combining Hyperbranched and Linear Structures in Solid Polymer Electrolytes to Enhance Mechanical Properties and Ion Transport

Benxin Jing[1,2,+], Xiaofeng Wang [3,+], Yi Shi[3], Yingxi Zhu [1,2*], Haifeng Gao[3*], and Susan K. Fullerton-Shirey[4,5*]

[1]Department of Chemical and Biomolecular Engineering, University of Notre Dame, Notre Dame, Indiana, United States

[2]Department of Chemical Engineering and Materials Science, Wayne State University, Detroit, Michigan, United States

[3]Department of Chemistry and Biochemistry, University of Notre Dame, Notre Dame, Indiana, United States

[4]Department of Electrical Engineering, University of Notre Dame, Notre Dame, Indiana, United States

[5]Department of Chemical and Petroleum Engineering, University of Pittsburgh, Pittsburgh, Pennsylvania, United States

1. Synthesis of HB PEO

<u>Inimer synthesis</u>. To generate the HB polymer core as the macroinitiator (MI), inimer BIEM, 2-(2-bromoisobutylryloxy) ethyl methacrylate, was synthesized according to a previously published method.[1-3] Briefly, a 100 mL round-bottom flask was loaded with a magnetic stir bar, 2-hydroxyethyl methacrylate (12.13 mL, 0.10 mol), pyridine (8.60 mL, 0.11 mol), and 70 mL dichloromethane (DCM), before cooled to 0 °C in an ice bath. 2-Bromoisobutyrl bromide (12.36 mL, 0.10 mol) dissolved in 15 mL DCM was added to the reaction mixture via addition funnel over 45 minutes. The reaction was kept cold for additional 45 minutes before being warmed up to room temperature for additional 3 hours. After reaction, the solvent was removed under reduced pressure and the product was purified with a silica column (4:1 hexanes: ethyl acetate), obtaining 25.68 g clear oil (92% yield). 500 MHz ^{1}H NMR spectroscopy was conducted using $CDCl_3$ as solvent showing the followings: δ=6.07 ppm (m, 1H,

$CH_2=C(CH_3)$), 5.52 ppm (m, 1H, $CH_2=C(CH_3)$), 4.35 ppm (m, 4H, OCH_2CH_2O), 1.88 ppm (s, 3H, $CH_2=C(CH_3)$, and 1.86 ppm (s, 6H, $C(CH_3)_2Br$).

HB MI synthesis. The MI was synthesized in microemulsion via activator generated by electron transfer (AGET) atom transfer radical polymerization (ATRP) of inimer**.** A typical procedure on polymerization of inimer with $[inimer]_0/[CuBr_2]_0/[dNbpy]_0/[sodium\ ascorbate]_0 = 70/1/2/0.5$ is briefly described. In a disposable test tube, dNbpy (10.6 mg, 0.026 mmol) and $CuBr_2$ (2.9 mg, 0.013 mmol) were mixed in 0.5 mL of DCM at 40 °C for half an hour before addition of BIEM inimer (0.25g, 0.90 mmol) to the mixture. After the evaporation of DCM at 40 °C, the mixture was added dropwise to a solution of 1 g Brij98 in 12 g water over 30 minutes to form a transparent microemulsion. Meanwhile, the temperature was slowly increased to 65 °C and stabilized for about 10 minutes before the injection of 0.10 mL sodium ascorbate aqueous solution to reduce the Cu(II) species and initiate the polymerization. The reaction was stopped after 2 hours by exposure to air. The emulsion was disrupted by adding THF to separate the HB polymer by centrifugation. The collected HB polymer was purified by dissolving in THF and precipitating from methanol three times. The polymer was then dried in vacuum oven under 50 °C overnight before next step.

Synthesis of hyperstar HB-65nm-2k and HB-80nm-1k. In the second step, the bottle-brush PEO arms were grown from the HB MI via surface-initiated ATRP (SI-ATRP) of oligo(ethylene glycol) methyl ether methacrylate (OEGMA, M_n = 950 and 2000). Typically, HBMI (56 mg, containing about 0.2 mmol alkyl bromine), PMDETA (60 μL), toluene (5mL), and 4 g of monomer were added to a Schlenk flask. The mixture was sealed and deoxygenated by three freeze-pump-thaw cycles. In the frozen state, CuBr (22.5 mg) and $CuBr_2$ (4 mg) were added under protection of N_2 flow. The flask was then subjected to two additional freeze-pump-N_2 backfilling cycles before being thawed at room

temperature. Subsequently, the flask was immersed into a preheated oil bath of constant 45 °C to start the reaction. While a small fraction of samples was taken to determine the monomer conversion using NMR, the reaction was stopped at a certain OEGMA conversion by exposure to air. The obtained hyperstar solution was passed through an alumina column to remove copper. Subsequently, the hyperstar polymers were precipitated from hexane and purified by dissolving in DCM and re-precipitated from hexane for three times. Finally, the collected hyperstar polymers were dissolved in benzene and lyophilized.

Synthesis of HB-106nm-1k-10k. To cap hyper-star PEO with a linear chain L10k, hyperstar with bottle-brush arms was made from oligo(ethylene glycol) methyl ether acrylate (OEGA, M_n = 1000) before replacing the terminal secondary alkyl bromine with azido group and coupling with alkyne-terminated L10k. HB-106nm-1k-10k was synthesized in four steps as follows. The first and second steps were the same as the previous hyperstar synthesis except the use of OEGA (M_n = 1000) as monomer to yield HB-68nm-1k. The bromine end group was replaced by azido group via substitution reaction with sodium azide in dimethylformamide (DMF). For the substitution reaction, 0.5 g hyper-star was dissolved in 10 mL DMF and added with 0.2 g sodium azide under magnetic stir. The reaction was carried out for 48 hours under 40 °C before the sodium azide was removed by centrifuge. After purification via precipitation from hexanes, the azido functionalized hyperstar polymer was freeze-dried before next step. Finally, the alkyne-functionalized PEO (L10k, M_n = 10,000) synthesized according to a previously reported procedures[4] was introduced to the hyperstar via copper(I)-catalyzed azide-alkyne cycloaddition (CuAAC) reaction in DMF.[4] The reaction started upon being heated to 45 °C. Samples were taken for the GPC measurement and the reaction was stopped when the elution volume in GPC became constant.

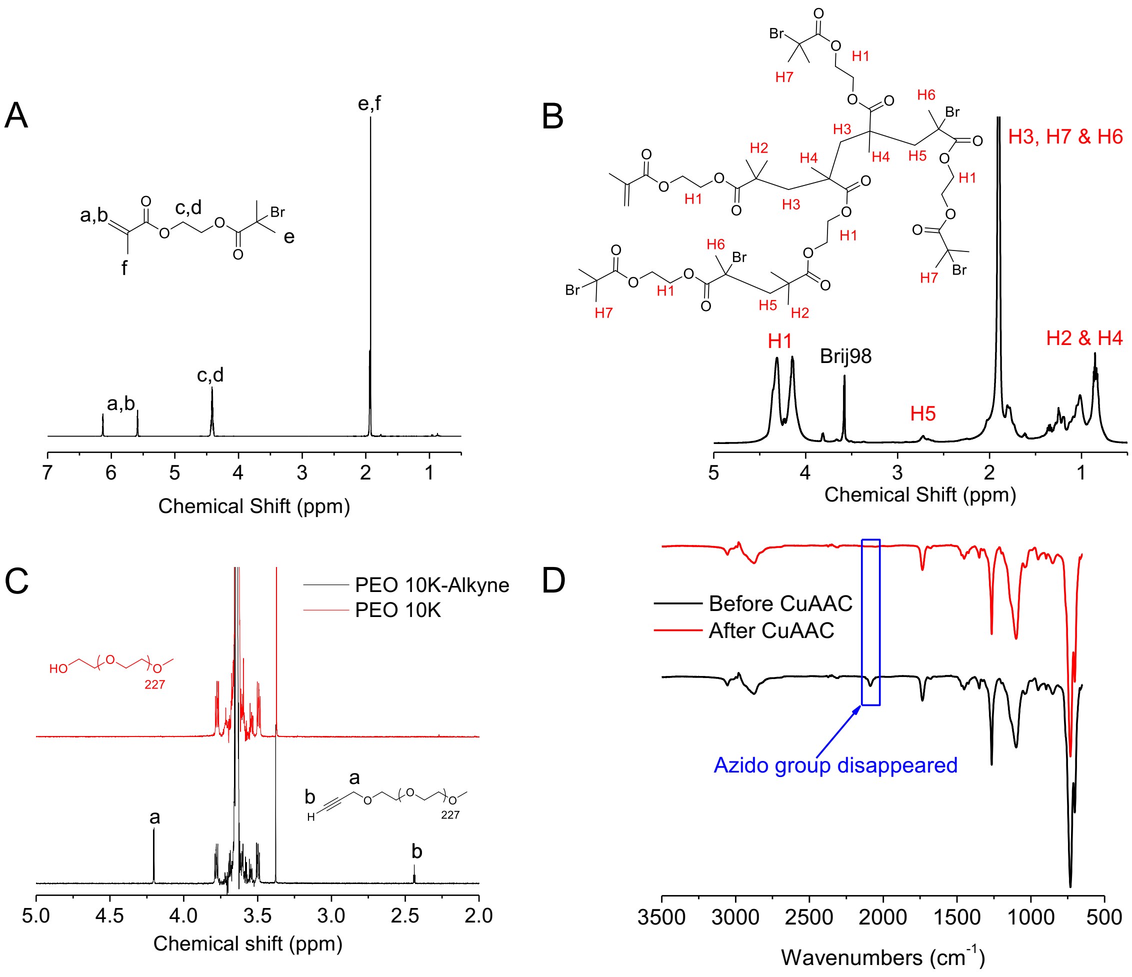

Supplementary Figure S1. ^{1}H NMR spectra of (A) inimer BIEM, (B) HB MI, (C) stacked ^{1}H NMR spectra of PEO 10k and PEO10k-Alkyne, and (D) FT-IR spectra of HB PEO before and after PEO 10k click reaction.

2. Representative force-distance curves

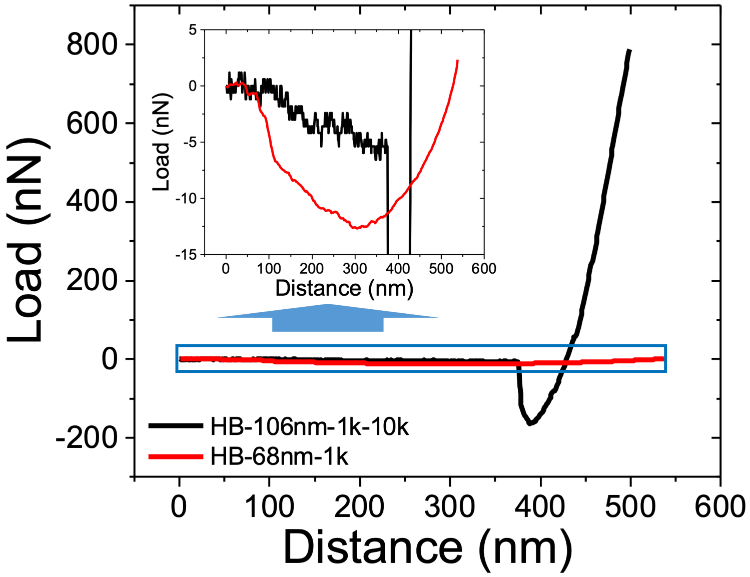

Supplementary Figure S2. Representative force-distance curves of PEO films without LiTFSI measured by nanoindentation. The spring constant of the AFM probe cantilevers are 44.39 N/m for HB-106nm-1k-10k film, and 0.50 N/m for HB-68nm-1k film.

3. AFM of linear chain PEO

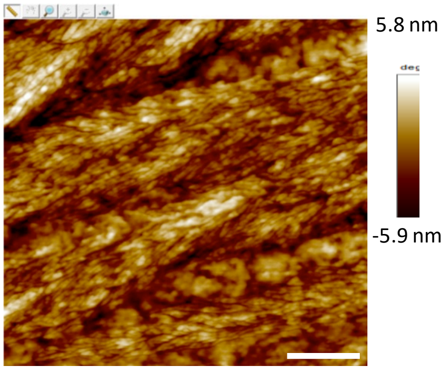

Supplementary Figure S3. AFM topology of the neat PEO L10k film (*i.e.*, without LiTFSI) by tapping mode. The scale bar is 400 nm.

4. Room temperature impedance data

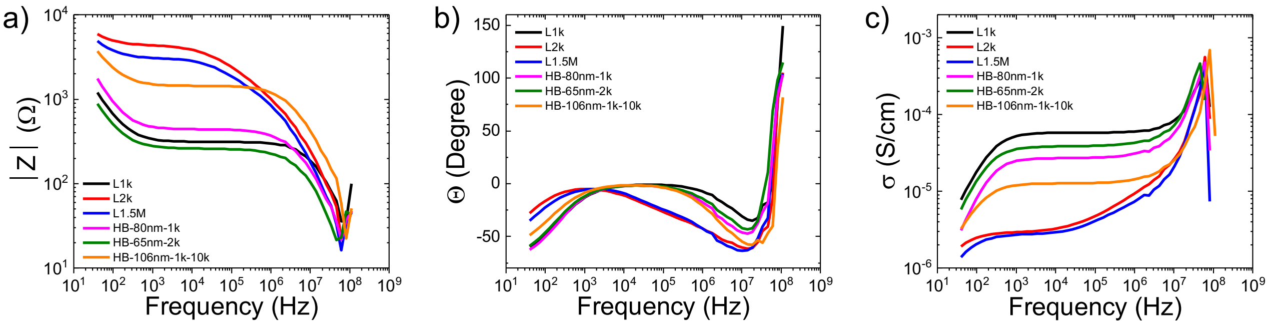

Supplementary Figure S4: a) Impedance, b) phase angle, and c) conductivity versus frequency at 22 ºC. Conductivity is calculated using equation (1) in the manuscript.

5. DSC measurements

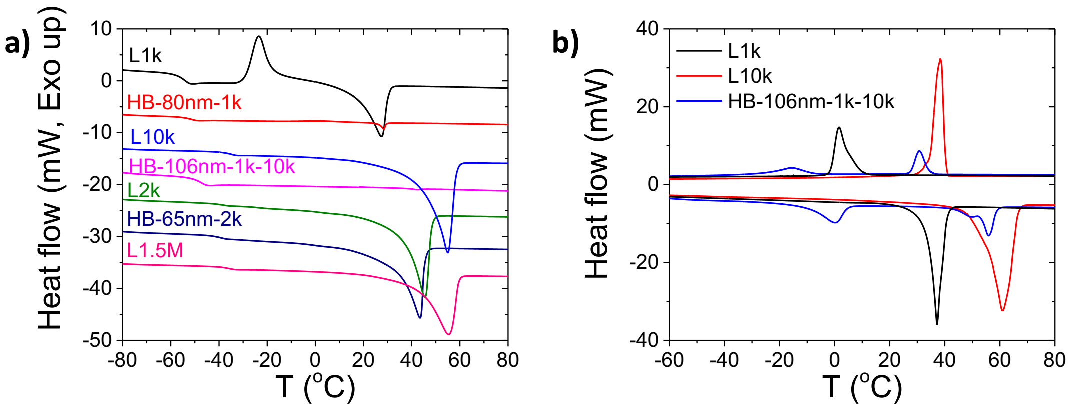

Supplementary Figure S5. DSC measurements. Measured heat flow versus temperature of (a) various PEO/LiTFSI electrolytes upon heating only and b) neat PEO (*i.e.*, without LiTFSI) upon both cooling and heating

REFFERENCES:

1. Matyjaszewski, K.; Gaynor, S. G.; Kulfan, A.; Podwika, M. Preparation of Hyperbranched Polyacrylates by Atom Transfer Radical Polymerization. 1. Acrylic AB* Monomers in "Living" Radical Polymerizations. *Macromolecules* **1997,** *30*, 5192-5194.

2. Graff, R. W.; Wang, X.; Gao, H. Exploring Self-Condensing Vinyl Polymerization of Inimers in Microemulsion To Regulate the Structures of Hyperbranched Polymers. *Macromolecules* **2015,** *48*, 2118-2126.

3. Min, K.; Gao, H. New Method To Access Hyperbranched Polymers with Uniform Structure via One-Pot Polymerization of Inimer in Microemulsion. *J. Am. Chem. Soc.* **2012,** *134*, 15680-15683.

4. Shi, Y.; Wang, X.; Graff, R. W.; Phillip, W. A.; Gao, H. Synthesis of Degradable Molecular Brushes via A Combination of Ring-opening Polymerization and Click Chemistry. *J. Polym. Sci., Part A: Polym. Chem.* **2015,** *53*, 239-248.
